# Targeted Modification and Structure-Activity Study of GL-29, an Analogue of the Antimicrobial Peptide Palustrin-2ISb

**DOI:** 10.3390/antibiotics11081048

**Published:** 2022-08-03

**Authors:** Siyan Liu, Yaxian Lin, Jiachen Liu, Xiaoling Chen, Chengbang Ma, Xinping Xi, Mei Zhou, Tianbao Chen, James F. Burrows, Lei Wang

**Affiliations:** School of Pharmacy, Queen’s University Belfast, 97 Lisburn Road, Belfast BT9 7BL, UK; sliu25@qub.ac.uk (S.L.); ylin36@qub.ac.uk (Y.L.); jliu49@qub.ac.uk (J.L.); c.ma@qub.ac.uk (C.M.); x.xi@qub.ac.uk (X.X.); m.zhou@qub.ac.uk (M.Z.); t.chen@qub.ac.uk (T.C.); j.burrows@qub.ac.uk (J.F.B.)

**Keywords:** antimicrobial peptides, palustrin-2, structure–activity relationships

## Abstract

Antimicrobial peptides (AMPs) are considered as promising antimicrobial agents due to their potent bioactivity. Palustrin-2 peptides were previously found to exhibit broad-spectrum antimicrobial activity with low haemolytic activity. Therefore, GL-29 was used as a template for further modification and study. Firstly, the truncated analogue, GL-22, was designed to examine the function of the ‘Rana box’, which was confirmed to have no impact on antimicrobial activity. The results of antimicrobial activity assessment against seven microorganisms demonstrated GL-22 to have a broad-spectrum antimicrobial activity, but weak potency against *Candida albicans* (*C. albicans*). These data were similar to those of GL-29, but GL-22 showed much lower haemolysis and lower cytotoxicity against HaCaT cells. Moreover, GL-22 exhibited potent in vivo activity at 4 × MIC against *Staphylococcus aureus* (*S. aureus*)-infected larvae. Several short analogues, from the C-terminus and N-terminus of GL-22, were modified to identify the shortest functional motif. However, the results demonstrated that the shorter peptides did not exhibit potent antimicrobial activity, and the factors that affect the bioactive potency of these short analogues need to be further studied.

## 1. Introduction

The discovery of antibiotics is considered as a milestone in medicine development and countless lives were saved in the 20th century as a result. However, the misuse and overuse of these agents has led to the rapid appearance of resistant strains, and as the World Health Organization reports, antibiotic resistance has become a primary crisis in global public health [1]. Current antibiotics have to be used prudently to hinder the growth of new drug-resistant bacteria and protect a drug’s efficacy [2]. Moreover, to avoid the desperate situation where no effective antibiotics are available, the development of alternative antimicrobials is of great importance. As an emerging field of research, antimicrobial peptides (AMPs) are considered to have potential as alternative antimicrobials as they display potent antimicrobial activity against bacterial pathogens mainly via non-targeted membrane permeabilisation and some peptides have even exhibited activities through multiple modes of action, which should hopefully make it difficult for bacteria to develop resistance against them [3].

Until now, a large number of natural AMPs have been discovered and reported to have both in vivo and in vitro antimicrobial, antibiofilm, anti-inflammatory and wound healing activities [4]. Amphibians are critical for the discovery of AMPs since anurans (frogs and toads) account for the largest proportion of AMP sources and structures, according to the Antimicrobial Peptide Database (APD). Numerous bioactive compounds have been discovered in amphibian skin secretions, which are a crucial protective mechanism for these creatures [5,6]. Although three different types of glands widely exist in amphibian skin, granular glands are of paramount importance to produce a wide variety of chemical compounds to protect the creature from being infected by bacteria and fungi [6] and the secretions contained within these glands can be harvested using mild electrical stimulation without harming the animals [7]. Cationic cytolytic peptides are not found in the skin secretions of all frogs, but they are widely distributed in species belonging to several anuran families, especially the Ranidae and Hylidae [8]. Antimicrobials from ranid frogs have been divided into several families according to their amino acid sequence similarities, including brevinin-1, brevinin-2, esculentin-1, esculentin-2, ranalexin, ranatuerin-1, ranatuerin-2, temporin, palustrin, tigerinin, japonicin-1, japonicin-2 and nigrocin-2 [9]. Additionally, the C-terminal cystine-bridged cyclic heptapeptide or hexapeptide called the ‘Rana box’, is highly conserved in antimicrobial peptides sourced from the Ranidae family [10].

In the palustrin-2 family, most peptides exhibit a broad-spectrum antimicrobial activity against Gram-positive and Gram-negative bacteria but little activity towards the yeast, *Candida albicans* (*C. albicans*) [11,12]. Additionally, they exhibit weak haemolytic activity which means they could be considered as potential alternatives to antibiotics [13]. Therefore, palustrin-2 peptides deserve more in-depth study. Peptides in this family mostly consist of 31 amino acids including a cyclic heptapeptide which is normally rich in glycyl residues, commonly followed by two C-terminal prolyl-containing amino acid residues, although some palustrin-2 peptides lack the last two residues such as palustrin-2ISa, palustrin-2ARa and palustrin-2VLa [8]. Moreover, palustrin-2ISb is unique compared to other palustrin-2 peptides as it consists of 36 amino acid residues with seven extra C-terminal residues, and although this peptide was isolated from *Odorrana ishikawae* (*O. ishikawae*), it exhibits higher sequence similarity to the palustrin-2 peptides isolated from *Odorrana grahami* such as palustrin-OM, palustrin-OG1, and palustrin-OG2, rather than those isolated from *O. ishikawae* [14].

Palustrin-2ISb (GLWNSIKIAGKKLFVNVLDKIRCKVAGGCKTSPDVE) was identified and previously examined for antimicrobial activities against four microorganisms, and an analogue of palustrin-2ISb, palustrin-2ISb-des-C7 (GLWNSIKIAGKKLFVNVLDKIRCKVAGGC), was synthesised by deleting the C-terminal 7 amino acids. The results showed that this short analogue had better antimicrobial activity than palustrin-2ISb as the MIC values of palustrin-2ISb against *Staphylococcus aureus* (*S. aureus*), *Escherichia coli* (*E. coli*) and methicillin-resistant *S. aureus* (MRSA) were 6.3 μM, 6.3 μM and 100 μM, respectively, whereas the results for palustrin-2ISb-des-C7 were 6.3 μM, 1.6 μM and 12.5 μM, respectively. Palustrin-2ISb-des-C7 also exhibited activity against *C. albicans*, with an MIC value of 100 μM, whilst, at the same concentration, palustrin-2ISb had no activity. Therefore, the short analogue palustrin-2ISb-des-C7 was chosen as the template in this study, and named GL-29 [14].

The conserved C-terminal motif, the ‘Rana box’, has been previously identified to be crucial for some peptides, such as ranalexin, where disruption of the Rana box led to a considerable reduction in antimicrobial activity [15]. However, for other peptides, the removal of the Rana box had no effect on antimicrobial activity. For instance, in nigrocin-HL and brevinin-2GHk, the removal of the Rana box had no significant effect on its antimicrobial activities, even decreased its haemolytic activity [16,17]. In addition, the Rana box in brevinin1E, only affects the formation of helical structure, but exerts no effects on bioactivity and the migration of the Rana box from the C-terminal to the middle part of the peptide, markedly reduced haemolysis with no effect on antibacterial activity [18,19]. Hence, a peptide lacking this motif, named GL-22 (GLWNSIKIAGKKLFVNVLDKIR-NH2) was synthesised and studied here.

As the expense of production is one of the largest barriers in the development of peptides for therapeutic applications, the truncation of peptides to active cores has been considered as a rational modification method to make them more cost-effective [20]. In addition, ultra-short peptides also have better tissue penetration and lower immunogenicity and toxicity [21,22]. In previous studies, short analogues truncated from long α-helical peptides, were proven to maintain potent antimicrobial activity, such as Pap12-1 from papiliocin, GGN5-N13 from gaegurin 5, and KR12 from LL-37 [23,24,25]. Glycine and proline residues in peptides can induce a kink in the middle of theα-helix to form a flexible region, which is significant for the orientation of the helix. This could also be helpful for membrane disruption in peptides which act via the carpet model [26,27]. Gaegurin 4 has two α-helical regions from residues 2–10 and residues 16–32, and these two helices are linked by a flexible loop region that is enhanced by the existence of two glycine residues. In addition, ranatuerin-2CSa also possesses two helical domains as the result of disruption by a turn structure caused by a threonine (Thr) residue [28,29]. Both of these peptides have a C-terminal rana box and some residues in the middle that resist the formation of helix structures. Thus, GL-22 could be suspected to have two helical parts since the prediction of secondary structure indicated the α-helical structure of GL-22 was disrupted by Gly10, Lys11 and Lys12, which may form a flexible structure in the middle of the peptide. Thus, in order to design a shorter peptide and investigate if one part of the helical structure could have bioactivity, or only the intact amphipathic helix could possess antimicrobial activity, a series of peptides was designed from GL-22 to determine its minimal functional unit.

## 2. Results

### 2.1. Rational Design, Prediction of Secondary Structure and Physicochemical Properties

A series of truncated analogues of palustrin-2ISb were studied in the following assays. At first, peptide GL-29, modified by deleting the C-terminal seven amino acids of palustrin-2ISb, was found to have similar antimicrobial activity to its parent peptide against *S. aureus*, *E. coli*, MRSA and *C. albicans*. The bioactive potency of GL-29 was further studied and the formation of the cyclic disulphide bridge in the ‘Rana box’ of palustrin-2ISb was found to have little influence on antimicrobial activity [30]. Therefore, a truncated peptide (GL-22), lacking the Rana box, was designed and studied. The N-terminal and C-terminal domains were predicted form two helical segments in GL-22 (Supplementary Materials Figure S1). Accordingly, a series of truncated analogues of GL-22 (GLWNSIKIAGKKLFVNVLDKIR-NH_2_) was designed and synthesised to learn if short segments of this peptide could exert proper antimicrobial potency. The N-terminal GL-9 (GLWNSIKIA-NH_2_) was produced as residues at the N-terminus of intact AMPs were reported to be critical for function in previous studies [31,32]. However, the results revealed a reduction in antimicrobial potency. Hence, to identify if the C-terminus of GL-22 was important, several analogues, LF-10 (LFVNVLDKIR-NH_2_), FV-9 (FVNVLDKIR-NH_2_), VN-8 (VNVLDKIR-NH_2_) and FV-8 (FVNVLDKI-NH_2_), were designed to determine which region was important for the potency of these peptides [25,33].

The secondary structures and 3D models of the peptides were predicted by PEP-FOLD 3 (https://bioserv.rpbs.univ-paris-diderot.fr/services/PEP-FOLD3/, accessed on 10 March 2021) and all the peptides were predicted to have a helical structure (Figure S1). GL-29 and GL-22 were predicted to have two main helical domains linked by several amino acids in the middle. The helical wheel plots (Supplementary Materials Figure S2) and physiochemical parameters were obtained from Heliquest (https://heliquest.ipmc.cnrs.fr/cgi-bin/ComputParams.py, accessed on 05 June 2021). The predicted physiochemical parameters of the peptides are listed in Table 1. GL-29 and GL-22 had similar hydrophobicity and charge as GL-22 has an extra C-terminal amidation. The results in Figure S1 showed that GL-9 could not really form a helical structure for the whole sequence, but LF-10 and FV-9 preferred to form a helix. Moreover, as shown in Table 1, the hydrophobicity of LF-10 and GL-9 was higher than FV-9 but they had the same net positive charge.

### 2.2. Synthesis, Purification and Identification of GL-29 and Its Analogues

The peptides were synthesised by solid-phase peptide synthesis (SPPS) and then purified by reverse-phase high-performance liquid chromatography (RP–HPLC) and each fraction was analysed by matrix-assisted laser desorption/ionisation time-of-flight mass spectrometry (MALDI-TOF MS). The chromatography results and mass spectra of GL-29 and its analogues are shown in Figure S3 and Figure S4, respectively. The molecular masses of the major single charged ions [M + H]^+^ were coincident with the predicted ones, confirming these peptides had been successfully synthesised.

### 2.3. Secondary Structures Analysis

**Figure 1:**
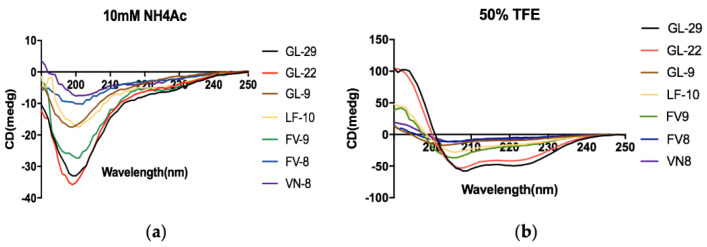
The CD spectra of GL-29 and its analogues in (**a**) 10 mM NH_4_Ac buffer (an aqueous environment) and (**b**) 50% TFE (mimicking the hydrophobic environment of the microbial membrane), respectively.

The circular dichroism (CD) spectra of peptides in two different solutions, 10 mM ammonium acetate (NH_4_Ac) (an aqueous environment) and 50% trifluoroethanol (TFE) (mimicking the hydrophobic environment of the microbial membrane), are shown in Figure 1. The results demonstrated that the two long peptides, GL-22 and GL-29, each adopted α-helical structures in the membrane-mimetic solutions since they possessed the typical positive peak at 193 nm and negative peaks at 208 nm and 222 nm, while they formed a clear random coil conformation in the aqueous environment. The helix, antiparallel and turn contents (%) of each peptide in NH_4_Ac buffer and 50% TFE, were analysed through the website BeStSel (http://bestsel.elte.hu/index.php, accessed on 20 August 2021) and are shown in Supplementary Materials Table S1.

As for the short analogues, LF-10 and FV-9 possessed a relatively higher helix percentage in the 50% TFE solution compared to other three analogue peptides which did not form an obvious helical structure in this environment, and these results were similar to the predicted ones.

### 2.4. Antimicrobial Assays and Determination of Cytotoxicity

#### 2.4.1. Antimicrobial Assays

The antimicrobial activities of GL-29 and its analogues are summarised in Table 2. In general, GL-29 and GL-22 showed broad-spectrum and significant antimicrobial potency against both Gram-positive and Gram-negative bacteria, including drug-resistant pathogens, while exhibiting weak activity against the yeast. Specifically, GL-29 showed a slightly better antimicrobial potency. Unfortunately, compared to GL-22, the short analogues, LF-10, FV-9, FV-8 and VN-8, showed almost no bioactivity, and only GL-9 showed antimicrobial activity at 512 μM. In terms of clinical isolates and drug-resistant strains, GL-29 and GL-22 exhibited similar activities against most tested strains compared to reference strains, but even better against MRSA (B038 V1S1 A) and *Klebsiella pneumoniae* (*K. pneumoniae*) (ATCC BAA-1705). However, the MIC of GL-22 against clinical *S. aureus* strain (B042 V2E1 A) showed an eight-fold decrease compared with the reference strain. Moreover, all short peptides had no antimicrobial activities against these strains under tested concentrations.

#### 2.4.2. Determination of Haemolytic Activity

**Figure 2:**
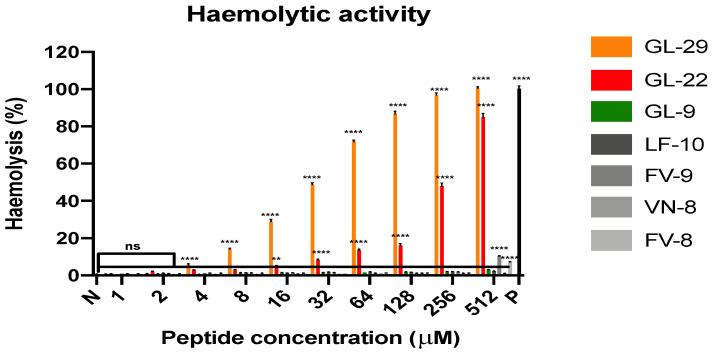
The haemolytic activity of GL-29 and its analogues at various concentrations from 1 to 512 µM on horse erythrocytes. P and N represent the positive control (0.1% TritonX-100) and negative control (PBS), respectively. The error bar represents means with SEM (standard error of mean) of nine repeats from three independent tests. The statistical significance was analysed using two-way ANOVA and is indicated as ns (non-significant difference), ** (*p* < 0.01), **** (*p* < 0.0001).

Concerning the haemolytic activity towards horse erythrocytes, GL-22 exhibited a considerable advantage, with an HC_50_ value larger than that of GL-29, by nearly eight-fold, as shown in Table 2, although they only showed slight differences on antimicrobial potency. Additionally, the short analogues exhibited almost no haemolysis even at high concentrations, except for FV-9 and FV-8, with HC_10_ values of 504.2 μM and 583.6 μM, respectively (Figure 2 and Table 2).

#### 2.4.3. Determination of Antiproliferative Activity on HaCaT

**Figure 3:**
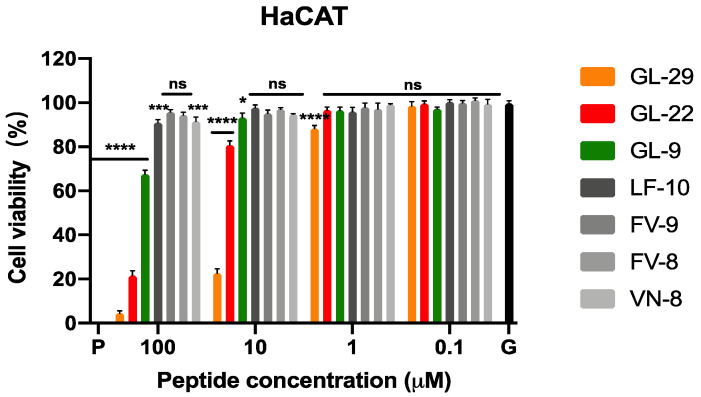
The effects of GL-29 and its analogues on the proliferation of the human keratinocyte cell line, HaCaT. P and G represent the positive control (cell growth with 0.1% TritonX-100) and growth control (cell growth without peptides), respectively. The percentage of cell viability was calculated by comparison with the growth control group. The error bars represent SEM of nine repeats from three independent tests. The statistical significance was analysed using two-way ANOVA by comparison with the growth control group and is indicated as ns (non-significant difference), * (*p* < 0.05), *** (*p* < 0.001), **** (*p* < 0.0001).

The MTT assay was conducted to evaluate the cytotoxicity of GL-29 and its analogues against human keratinocytes (Figure 3). GL-29 showed strong cytotoxicity against this cell line even at low concentration with an IC_50_ value of 5.05 μM. However, GL-22 demonstrated a relatively lower cytotoxic effect as it only inhibited 20% cell growth at 10 μM, and the IC_50_ value of GL-22 showed around an eight-fold increase compared to GL-29. In addition, the IC_50_ value of GL-22 was higher than its MIC values against most tested bacterial strains. In regard to those short analogues, they only inhibited modest effect on the inhibition of cell growth even at the highest tested concentration.

### 2.5. Antibiofilm Activity of GL-29 and Its Analogues

The results of antibiofilm assays (Table 3) showed that GL-22 had no ability to eradicate bacterial biofilms and GL-29 could only eradicate biofilms of three bacteria at a high peptide concentration and both of them showed a weaker inhibitory activity towards formation of Gram-negative bacterial biofilm, especially *P. aeruginosa* (ATCC CRM 9027). However, the concentrations of peptide required to inhibit formation of bacterial biofilms were mostly consistent with their MIC values. As for the short analogues, they exhibited nearly no ability to inhibit the growth of biofilm, which was consistent with their antimicrobial activity.

### 2.6. Membrane Permeability Assays and Fluorescence Microscopy

**Figure 4:**
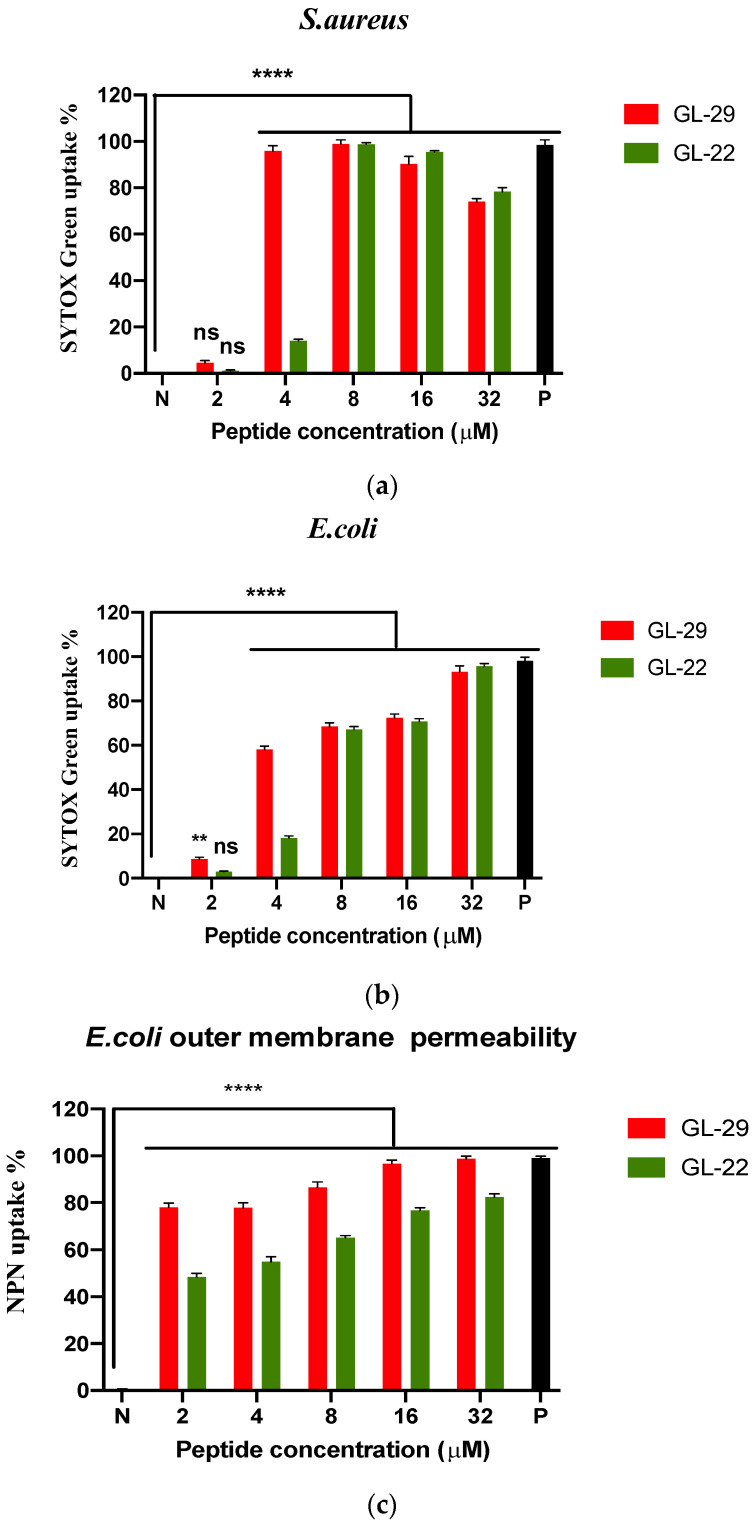
The membrane permeabilisation of (**a**) *S. aureus* (ATCC CRM 6538) and (**b**) *E. coli* (ATCC CRM 8739) and (**c**) outer membrane permeabilisation of *E. coli* (ATCC CRM 8739) with GL-29 and GL-22 at concentrations of 2 μM, 4 μM, 8 μM, 16 μM and 32 μM. P and N represent positive control and negative control. Melittin (16 μM) was employed as a positive control. The negative control was bacterial cell suspension with no treatment. The percentage of permeability was calculated by comparison with the negative control group. The error bars represent means with SEM of nine repeats from three independent tests. The statistical significance was analysed using two-way ANOVA by comparison with the negative control group and is indicated as ns (non-significant difference), ** (*p* < 0.01), **** (*p* < 0.0001).

**Figure 5:**
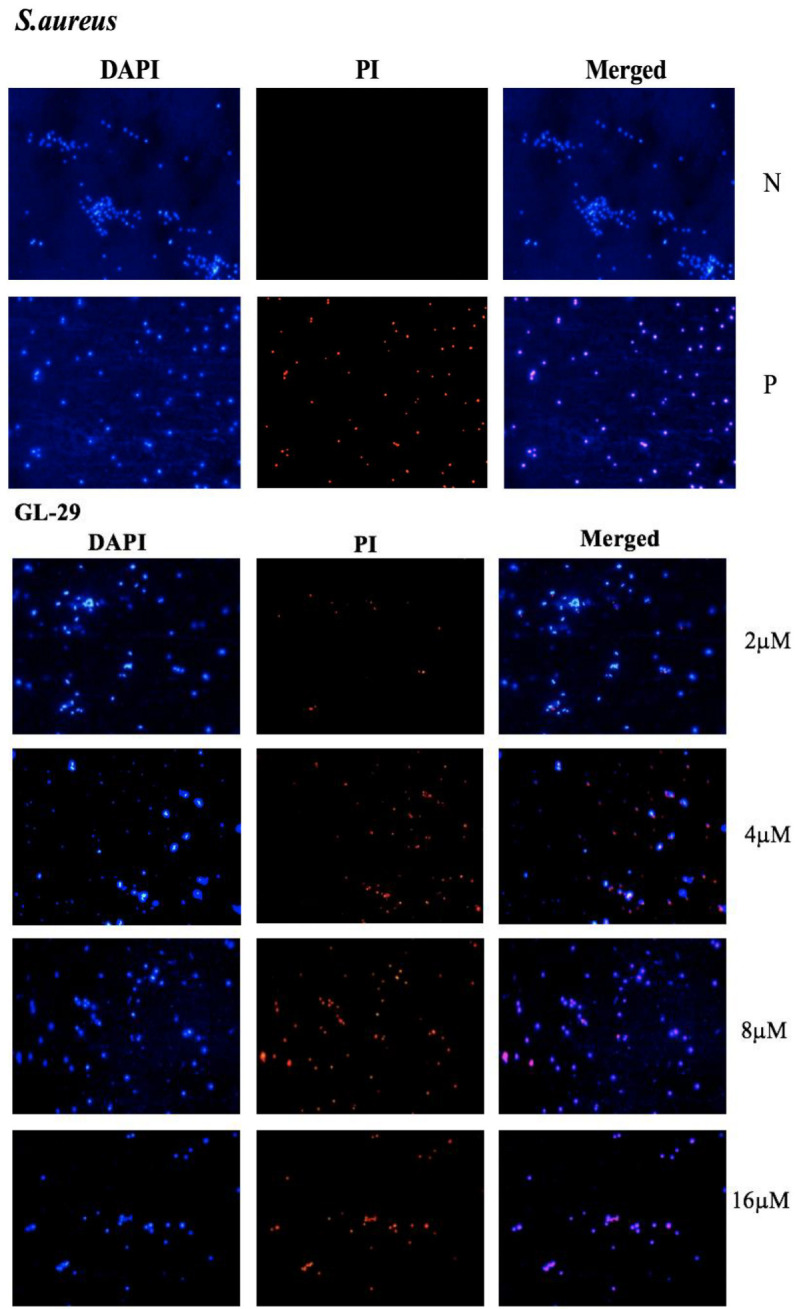

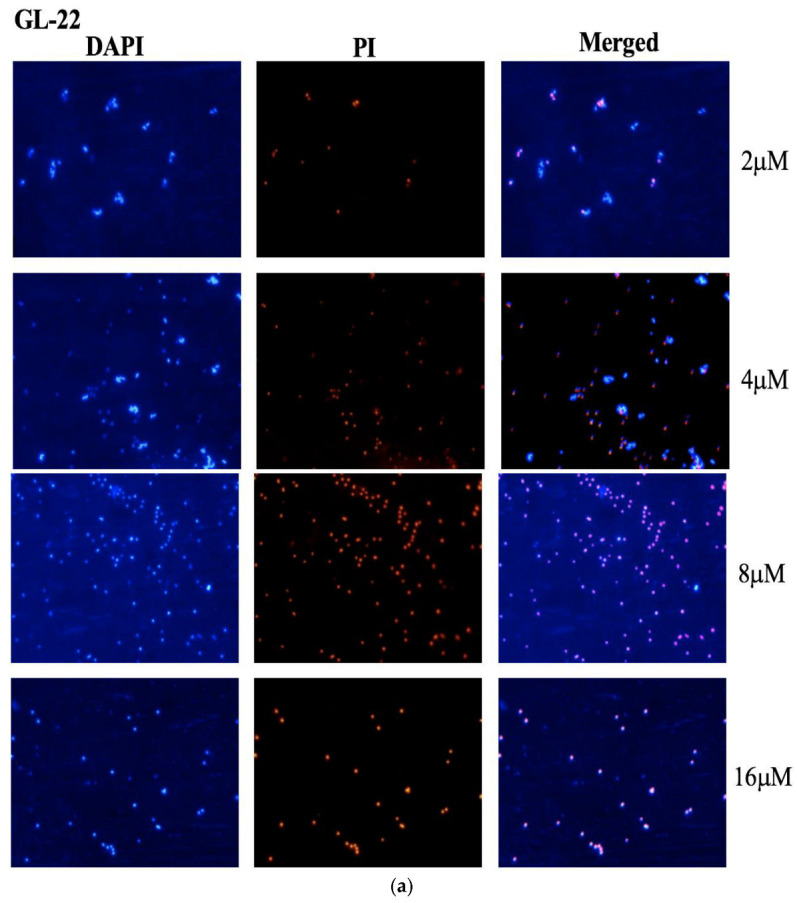

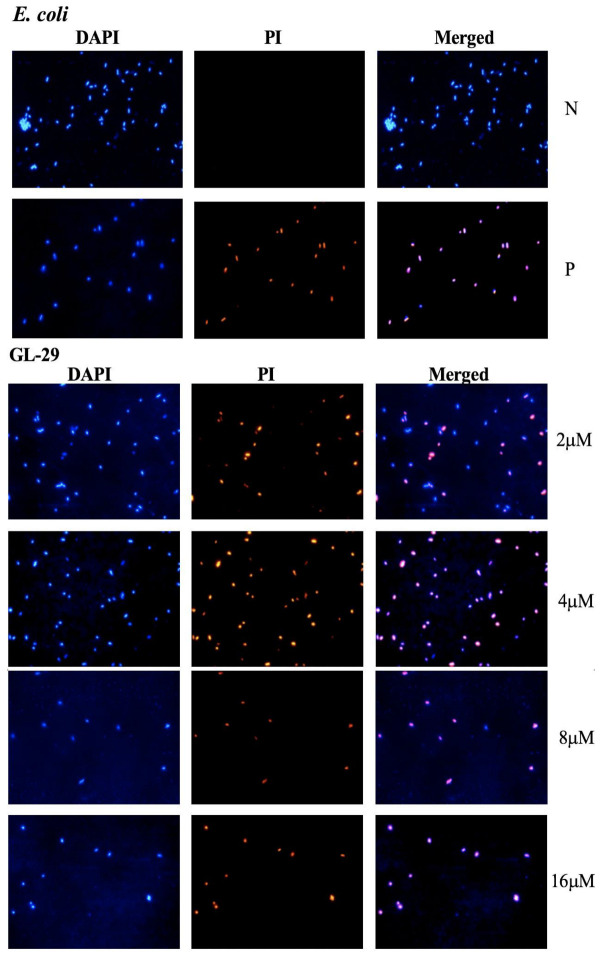

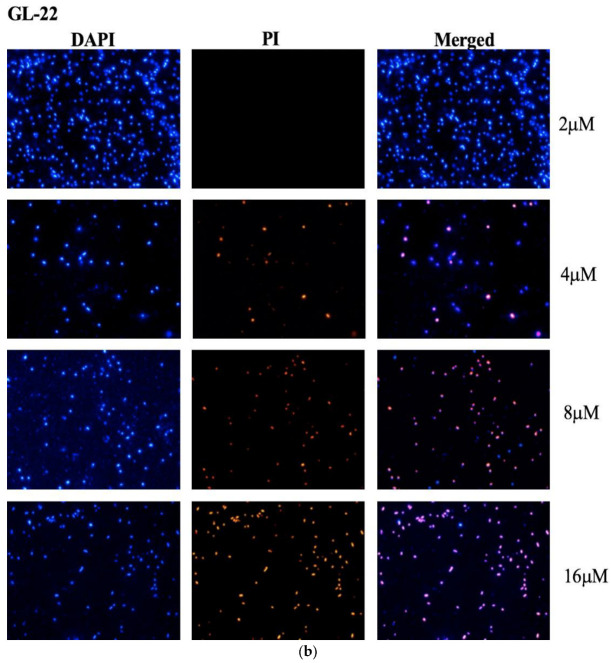
Fluorescence micrographs of DAPI/PI staining (**a**) *S. aureus* (ATCC CRM 6538) and (**b**) *E. coli* (ATCC CRM 8739) treated with GL-29 and GL-22 at different concentrations. N and P represent the negative control and positive control, respectively. Melittin (16 μM) was employed as a positive control. The negative control was bacterial cell suspension with no treatment.

The mechanism of GL-22 and GL-29 antimicrobial activity against *S. aureus* (ATCC CRM 6538) and *E. coli* (ATCC CRM 8739) was then studied. Bacterial membrane permeability assays were conducted using SYTOX Green Nucleic Acid Stain uptake and the permeabilisation elicited by these peptides was determined in a series of concentrations from 2 μM to 32 μM (Figure 4). Moreover, all bacterial cells were observed by fluorescence microscopy after staining with 4′,6-diamidino-2-phenylindole (DAPI), whereas only cells with permeabilised membranes stained with propidium iodide (PI). The outer cell membrane permeability of Gram-negative bacteria was tested using N-Phenyl-1-naphthylamine (NPN) uptake assay at the same concentrations. The permeabilisation of GL-29 against *S. aureus* reached 100% by 4 μM, but the percentage was only 20% for GL-22, and the DAPI/PI staining assay results were mostly consistent with the SYTOX green stain uptake assay, except that GL-22 permeabilised nearly 50% of bacterial membranes (Figure 5), which revealed that GL-29 exhibited a little higher permeability than GL-22 at 4 μM but in higher concentration they both showed nearly 100% permeabilisation. Differing from the SYTOX green stain uptake assay, the DAPI/PI staining results (Figure 5) demonstrated that these two peptides permeabilised the *E. coli* cell membrane completely at concentrations above 4 μM. Moreover, the outer membrane of *E. coli* was mostly permeabilised at very low concentrations of both peptides (Figure 4c).

### 2.7. Antiproliferative Activity Assays

**Figure 6:**
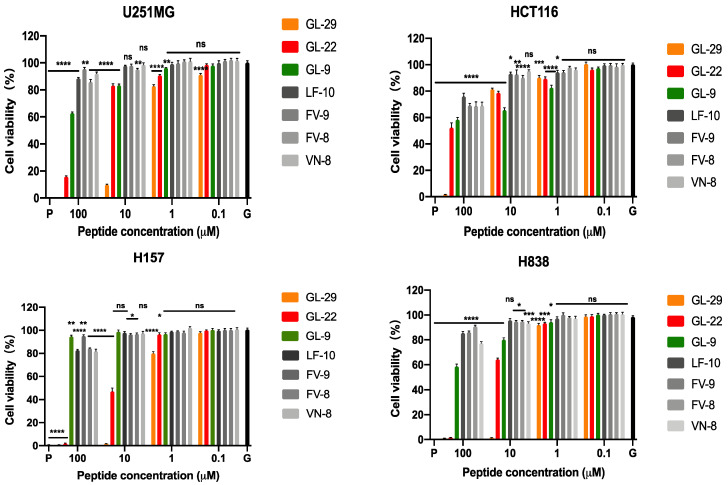
The effects of GL-29 and its analogues on the proliferation of the human malignant glioblastoma astrocytoma cell line, U251MG, human colon cancer cell line, HCT116, human non-small lung cancer cell line, H157 and H838. P and G represent the positive control (cell growth with 0.1% TritonX-100) and growth control (cell growth without peptides), respectively. The percentage of cell viability was calculated by comparison with the growth control group. The error bars represent SEM of nine repeats from three independent tests. The statistical significance was analysed using two-way ANOVA by comparison with the growth control group and is indicated as ns (non-significant difference), * (*p* < 0.05), ** (*p* < 0.01), *** (*p* < 0.001), **** (*p* < 0.0001).

GL-29 displayed a more notable effect than GL-22 on cell viability of all the cell lines examined (Table 4). Both peptides showed a relatively low antiproliferative activity against a human colon cancer cell line, HCT116, and they all had higher potency against human lung cancer cell lines (H157, H838). In keeping with their antimicrobial potencies, the short analogues exhibited very weak activity against the proliferation of the cells generally. GL-9 could inhibit around 20–40% of proliferative activity of the three cancer cell lines, U251MG, HCT116 and H838, at 100 µM (Figure 6). The other four short analogues had virtually no antiproliferative activity, even at the highest concentrations.

### 2.8. S. aureus-Infected Larvae Treatment In Vivo

**Figure 7:**
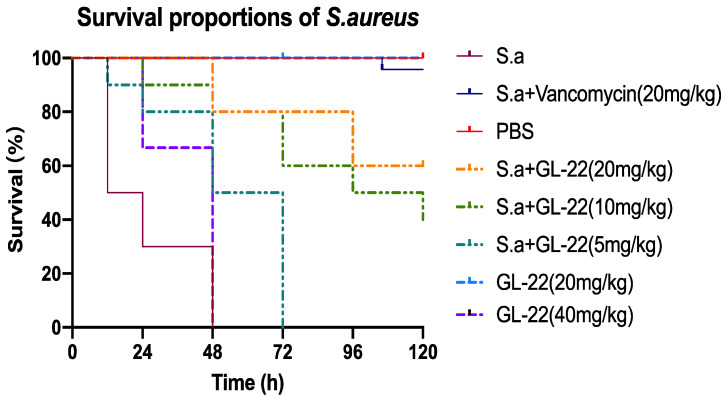
The survival percentage of waxworms (n = 9 each group) infected with *S. aureus* and treated with GL-22 at concentrations varying from 5 mg/kg to 20 mg/kg. The *S. aureus*-infected larvae without any treatment and treated with vancomycin (20mg/kg) were considered as the negative control and positive control, respectively. The larvae treated with PBS only, were seen as the vehicle control. The toxicity of peptide was tested to treat larvae merely with GL-22 (40 mg/kg) and GL-22 (20 mg/kg) separately.

GL-22 exhibited a higher selectivity than GL-29 since GL-22 showed a much lower haemolysis activity and cytotoxicity against HaCaT, maintaining similar antimicrobial activity in the meantime. Therefore, to further test the toxicity of GL-22, an in vivo assay was conducted (Figure 7). The results show GL-22 had high toxicity at 4 × MIC (40 mg/kg) against larvae, but it exhibited no toxicity at 2 × MIC (20 mg/kg) at all. Therefore, *S. aureus*-infected larvae were treated with GL-22 at 2 × MIC (20 mg/kg), 1 × MIC (10 mg/kg) and 1/2 × MIC (5 mg/kg). Compared to the control group, the mortality of *S. aureus*-infected larvae treated with GL-22 at 1 × MIC (10 mg/kg) was notably lower, something which was further improved when 2 × MIC (20 mg/kg) was used. This indicated the peptide was able to limit the mortality caused by the *S. aureus* infection.

## 3. Discussion

Antimicrobial peptides have been determined to be potential antibiotic alternatives which potentially avoid antibiotic resistance because they act through membrane permeabilisation or multiple modes of action [34]. In the palustrin-2 family, most peptides exhibit broad-spectrum antimicrobial activity against Gram-positive and Gram-negative bacteria, but not against the yeast, *C. albicans* and the Gram-negative bacterium, *P. aeruginosa*. They also show low haemolytic activity against red blood cells [11,12]. These properties led us to choose GL-29 as the subject of this study.

The conserved C-terminal motif, the ‘Rana box’, has been found to be unrelated to the antimicrobial activity of nigrocin-HL and brevinin-2GHk, and the removal of this ‘Rana box’, also decreased their haemolysis [16,17]. The ‘Rana box’ in brevinin1E was useful for the formation of a helical structure, but the disulphide bridge of the ‘Rana box’ exerts no effects on bioactivity [18]. Therefore, a truncated analogue of GL-29 without the ‘Rana box’ was designed and GL-22 was found to exhibit a similar bioactivity profile to GL-29, on both bacteria and fungi. This may be because the removal of this motif had no significant influence on the helicity, hydrophobicity and positive charge. The helical structure and hydrophobicity of GL-22 were similar to those of GL-29 (Figure 1, Table S1, Table 1), and the cationic amino acid in this motif was compensated for by the C-terminal amide. It is possible to conclude that the disulphide bridge of the ‘Rana box’ in GL-29 may have just a small effect on antimicrobial activity. However, the removal of this motif considerably reduced the haemolysis and cytotoxicity against HaCaT of this peptide and enhanced the selectivity accordingly. As with other palustrin-2 peptides, GL-22 and GL-29 were found to have broad-spectrum antimicrobial activity, except against *C. albicans,* and they showed relatively weaker activity against *P. aeruginosa*. The weak bioactivity on *C. albicans* may be due to the different components of *C. albicans* cell wall. Normally, the cell wall of a fungus is composed of chitin, which is not the main target of peptides and is more rigid compared to peptidoglycan, the main component of the bacteria cell wall [35,36]. In addition, the possible reason why GL-22 and GL-29 showed relatively lower activity toward *P. aeruginosa* was the different LPS types in the outer membrane of *P. aeruginosa,* which leads to an alternation of membrane surface characteristics, and this influences the electronic attraction and hydrophobic interaction of peptides toward this species [37]. Moreover, GL-29 and GL-22 exhibited strong potency against the two tested clinical strains, including the cystic fibrosis (CF) isolated strains, *S. aureus* (B042 V2E1 A) and MRSA (B038 V1S1 A) [31]. Moreover, these two peptides possessed extraordinary activities against KPC-producing strains, including *E. coli* (ATCC BAA-2340) and *K. pneumoniae* (ATCC BAA-1705), which made them valuable for further studies [38,39]. In addition, it is widely accepted that the antibacterial mechanism of AMPs is the membrane permeabilisation mode of action [40]. The result of permeability assays showed that GL-29 and GL-22 could kill the pathogens through membrane permeabilisation. According to previous studies, the process was speculated to have several steps, including the initial attraction and binding to membranes via electrostatic interaction, after which, peptides begin to accumulate to reach a threshold concentration to help them to exert action on the bacterial membranes, then, the amphipathic conformation and the hydrophobicity of peptides help them to insert into the cell membrane and cause cell death [41,42]. As the fluorescence microscopy (Figure 5) demonstrates, GL-22 caused more membrane permeabilisation of *S. aureus* than *E. coli* at 1/2 × MIC (2 μM). The possible reason was that the extra outer membrane of Gram-negative bacteria may prevent the peptides from attaching to the inner membrane [43]. Moreover, previous research has found that except for permeabilisation, some AMPs also have multiple modes of action, including interaction with cell wall or intracellular targets [44]. In detail, the cell wall or outer membrane in Gram-negative bacteria is essential for preventing cell lysis and anchoring membrane components; hence, it is possible for AMPs to bind with the main building block of peptidoglycan and lipopolysaccharide (LPS), which were essential components in cell wall of Gram-positive and outer membrane of Gram-negative bacteria, separately, and then cause damage to bacteria [36,45,46,47]. Moreover, some peptides (e.g., proline-rich antimicrobial peptides (PR-AMPs), PR-39 and Bac7 (1-35)) could traverse through cell barriers and interact with intracellular targets and then cause the inhibition of protein synthesis and inhibition of enzymatic activities [44,48,49]. As for the peptides studied here, the exact mechanisms for how they could exert antimicrobial potency through interaction with specific targets are unknown and need to be further studied.

Regarding the characteristics that affect AMPs’ functions, several features, including the conformation, charge, amphipathicity (hydrophobic moment) and hydrophobicity, were considered to play a predominant role [50]. Concerning the conformation, AMPs possess a wide range of secondary structures, in which α-helix and β-sheet play a predominant role in conferring activities [51]. The rearrangement of conformation of AMPs is a key process for interaction with biomembranes after attachment to the membrane, and this process was documented to occur in α-helical AMPs since most of them were observed to be disordered in an aqueous solution, but once interacted with lipid bilayers, they will assume a conformation transition to an amphipathic α-helix, such as magainin and PGLa [52,53,54]. Therefore, due to the importance of the helicity, additional truncated analogues of GL-22 were designed to find if the two helical segments (Supplementary Materials Figure S1) were active motifs themselves. According to the CD spectra (Figure 1) and calculated data (Table S1), GL-9, FV-8 and VN-8 could transfer from the disordered status in aqueous environment to the helical structure in membrane-mimic solution, but the percentage of the helix in these three analogues were obviously lower than that in GL-29, GL-22 and other two short analogues. In a previous study, the truncated N-terminal derivatives, which possessed similar helicities to the parent peptide, maintained antimicrobial activities, while those analogues with significantly reduced helical content usually lost bioactivities; hence, the decrease of helical content could therefore be a possible reason for the obvious reduction in antimicrobial activity for GL-9, FV-8 and VN-8 [25,55]. Moreover, the loss of antimicrobial potency and helicity could also be due to the very short length of the peptides, as normally, 12–13 residues are required for the retention of bioactivity when truncating larger parent peptides [23,25,56]. However, a shorter peptide, with only eight residues from the temporin family, was reported to have high potency against Gram-positive bacteria [20]. For example, peptides truncated from chensinin-1b, as short as nine residues, also possess some activity against both Gram-positive and Gram-negative bacteria [33], suggesting that the loss of bioactivity was not completely related to the short length. Additionally, cationic residues are of great importance as the electrostatic interaction between positively charged amino acids and the anionic components (e.g., membrane lipids) of cell surfaces lead to the initial attraction of AMPs to the pathogen cell surface [41,57]. Usually, cationic AMPs require at least +2 net charges to exert their bioactivity [55], while there was only one lysine in the short derivatives, and therefore the loss of bioactivity may be due to the decrease of net positive charge in the short derivatives. However, the peptides palustrin-2ISb-des-C7-des-N9 (GKKLFVNVLDKIRCKVAGGC), has one helical part of the peptide Pa2ISb-des-C7, with a high positive charge and was previously found to have little activity against Gram-positive and Gram-negative bacteria [30], which meant a high positive charge was probably not the main factor affecting antimicrobial activity for the C-terminal derivatives.

In addition to all the factors mentioned above, hydrophobicity is also crucial for antimicrobial activities due to hydrophobic interaction being essential in partitioning peptides into the lipid bilayers and it determines the extent of this process [34,52]. According to the results from this study, the higher hydrophobicity of GL-9 may be the main reason for its higher bioactive potency, as it has the same net charge as other analogues and has a lower amount of helix in contrast to LF-10 and FV-9. Hydrophobicity has been speculated to play a predominant role in the bioactive capabilities of these short peptides. Moreover, despite higher hydrophobicity of GL-9 and LF-10 as the predicted date shown in Table 1, the haemolytic activity of them was lower than that of FV-9 and FV-8, thus the phenylalanine (Phe) exposed at the N-terminus in the analogues FV-9 and FV-8 instead of hydrophobicity was supposed to be the prime culprit for the haemolysis for this series of short analogues. In addition, there is an aspartic acid (Asp) in all C-terminal analogues, and acidic residues that exist in the vicinity of basic residues have been reported to have a negative effect on anionic membrane perturbation. This is because the hydrophobic face is bordered by negative charges, which hinder the attraction of the peptide to the negatively charged lipids [58]. Consequently, one of the possible reasons for the ineffectiveness of these four peptides could be the incorrect position of the negatively charged amino acid.

Overall, the results showed that N-terminal and C-terminal sections exhibited very little, or no, antimicrobial activity. It is a possibility that the two sections have to work together, such as gaegurin 4, which permeabilises the membrane by having its N-terminal helix segment initially bind to the membrane then the C-terminal section forms ionic pores [59]. However, a more possible explanation of the cause of this phenomenon, is a combination of the lack of charge, hydrophobicity and helicity in these short analogues [25,31,60]. Therefore, the effects of these features should be further studied.

## 4. Materials and Methods

### 4.1. Synthesis, Purification and Identification

Each peptide was chemically synthesised via solid-phase Fmoc chemistry using a Tribute automated peptide synthesiser (Protein Technologies, Tucson, AZ, USA), which was described in the previous study [61]. Rink amide resin was employed as solid phase for the synthesis process. The synthetic peptides were released from the resin by adding a cleavage cocktail, which contains 94% trifluoroacetic acid (TFA), 2% water, 2% thioanisole (TIS), and 2% 1,2-ethanedithiol (EDT) for 2 h at room temperature and further washed by diethyl ether three times. After lyophilisation, the crude peptides were then purified by RP–HPLC (Phenomenex Aeris PEPTIDE 5 μm XB-C18 column, 250 mm × 21.2 mm, Macclesfield, Cheshire, UK) by gradient elution from 90% solution A (99.95% dd H2O and 0.05% TFA) and 10% solution B (80% acetonitrile, 19.95% dd H2O and 0.05% TFA) to 100% solution B at a flow rate of 8 mL/min. The purity of peptides was identified by RP–HPLC and MALDI-TOF mass spectrometry (Voyager DE, Perspective Biosystem, Foster City, CA, USA) in positive detection mode using CHCA (α-cyano-4-hydroxycinnamic acid) as the matrix.

### 4.2. Secondary Structure Prediction and Analysis

The secondary structures of peptides were predicted by use of the online tool PEP-FOLD 3 (https://bioserv.rpbs.univ-paris-diderot.fr/services/PEP-FOLD3/, accessed on 10 March 2021). Then, the Heliquest programme was used to predict physicochemical properties of peptides and the helical wheel plots of secondary structure (https://heliquest.ipmc.cnrs.fr/cgi-bin/ComputParams.py, accessed on 05 June 2021). Then, the secondary structures of the peptides were examined by a JASCO J-815 CD spectrometer (JASCO, Essex, UK). The peptide samples (100 μM) were dissolved in 10 mM NH4Ac solution and 50% TFE/NH4Ac (*v/v*), respectively. After the samples were loading in a 1-mm thickness quartz cuvette, they were analysed at room temperature with the scan range of 190–250 nm. The scanning speed was 100 nm/min with 1 nm bandwidth and 0.5 nm data pitch. The spectra were achieved by the average data of three scans.

### 4.3. MIC and MBC Assays

The antimicrobial activity of the peptides was determined through the minimum inhibitory concentrations (MICs) and the minimum bactericidal concentration (MBCs) using the broth-dilution method in a 96-well plate, as described in the previous study [61]. Six bacteria, including the Gram-positive bacteria, *S. aureus* (ATCC CRM 6538), MRSA (ATCC CRM 12493) and *E. faecalis* (NCTC 12697), the Gram-negative bacteria, *E. coli* (ATCC CRM 8739), *K. pneumoniae* (ATCC CRM 43816) and *P. aeruginosa* (ATCC CRM 9027), the yeast, *C. albicans* (ATCC CRM 10231) and clinical isolates, *S. aureus* (B042 V2E1 A), MRSA (B038 V1S1 A), *E. coli* (ATCC BAA-2340) and *K. pneumoniae* (ATCC BAA-1705) were used in these assays to evaluate the antimicrobial potency of the two peptides by determining their MICs and MBCs. All bacteria were cultured in MHB and the yeast, C. albicans was cultured in YPD broth overnight. Then, the microorganisms were sub-cultured and diluted to the final concentration of 2 × 10^5^ CFU/mL to 5 × 10^5^ CFU/mL. The Norfloxacin and amphotericin B were used as positive controls for the bacteria and yeast, respectively. The assay was repeated three times, with five replicates at each time.

### 4.4. MBIC and MBEC Assays

The MBIC and MBEC assays were performed to measure the inhibition and eradication of bacterial biofilm in the presence of different concentrations of peptides. Gram-positive bacteria were inoculated in Luria–Bertani (LB) broth at 37 °C overnight whereas Gram-negative bacteria were inoculated in tryptic soy broth (TSB), and then sub-cultured until reaching the logarithmic growth phase by measuring the OD values at a wavelength of 550nm. For the MBIC assay, 99 µL of diluted bacterial suspension (5 × 10^5^ CFU/mL) was mixed with 1 µL of prepared peptide solutions in the wells of a round-bottomed 96-well plate, achieving the final concentrations from 512 to 1 µM, and then incubated at 37 °C for 24 h in a shaking incubator at 200 revolutions per minute (rpm). After this, the plate was washed twice by PBS and fixed with methanol for 30 min. For MBEC assay, 100 µL of diluted bacterial suspension (5 × 10^5^ CFU/mL) was seeded into a round-bottomed 96-well plate and incubated at 37 °C for 24 h in a shaking incubator at 200 rpm to obtain mature biofilm. After this, plates were washed twice by PBS and incubated with different concentrations of peptide solution from 512 to 1 µM at 37 °C for 24 h. Afterwards, the plate was washed twice with PBS and fixed with methanol for 30 min. Then, both MBIC and MBEC plates were stained by 130 μL of 0.1% (*g/v*) crystal violet solutions for 30 min and washed with PBS until no apparent staining was observed. Evaporating crystal violet stains were dissolved by 33% (*v/v*) acetic acid, and the absorbance values were measured by a Synergy HT Microplate Reader (BioTek, Minneapolis, MN, USA) set to 595 nm. The minimum concentration that inhibited the formation of biofilm was defined when compared to the negative control group, as the MBIC. The minimum concentration that eradicated the biofilm was defined, when compared to the negative control group, as the MBEC. The biofilm assays were performed in triplicate.

### 4.5. SYTOX Green Dye Uptake Assays

Bacteria were inoculated into TSB medium (Gram-positive bacteria) or LB medium (Gram-negative bacteria) at 37 °C overnight, and then sub-cultured at 37 °C for about 2.5 h. Subsequently, the cell cultures were centrifuged at 1000× *g* at 4 °C for 10 min to collect bacteria cells at the bottom. After the cells were washed twice with 5% TSB or 5% LB in 0.85% NaCl solution, they were resuspended with 5% TSB or 5% LB in 0.85% NaCl solution to obtain a cell concentration of 1 × 10^8^ CFU /mL by measuring the optical density (OD) value at 590 nm (Gram-positive bacteria: 0.68–0.72, Gram-negative bacteria: 0.65–0.7). The cell suspensions were then mixed with peptides at a range of concentrations, according to the MIC of antimicrobial assays, in a black 96 well plate and incubated for 2 h at 37 °C. After the incubation, the cells were stained with SYTOX^TM^ Green Nucleic Acid Stain (Thermo Fisher Scientific, Waltham, MA, USA) dissolved in 5% TSB in 0.85% NaCl to a final concentration of 5 μM and placed in a shaking incubator for 5 min avoiding from light. Bacterial cells treated with 16 μM melittin were used as positive control. The fluorescence intensity was measured with a Synergy HT plate reader (BioTek, USA) at excitation and emission wavelengths of 485 and 528 nm, respectively.

### 4.6. NPN Outer Membrane Assays

The outer membrane permeability assay was performed by using NPN, a fluorescent dye that is sensitive to the outer membrane of Gram-negative bacteria. In this assay, *E. coli* was at first inoculated in LB at 37 °C overnight, and then sub-cultured at 37 °C to the logarithmic phase (OD_550_ = 0.42), after which the bacteria were transferred to a 50 mL centrifuge tube and centrifuged at 4 °C 1000× *g* for 10 min. The bacteria were then washed twice with 5 mM HEPES buffer containing 5 mM glucose (pH = 7.2) and resuspended to an OD_600_ value of 0.5. Afterwards, 100 μL of bacterial culture and 50 L of peptide solution, were mixed in each well of the black 96-well plate. The negative control was constituted by HEPES buffer and positive control was constituted by 16 μM melittin, and then the plate was incubated at 37 °C for 2 h. After removal from the incubator, 50 μL of NPN (final concentration of 10 μM in each well) were added and the dye was mixed by putting the plate in a shaking incubator for 5 min. The absorbance was measured with a Synergy HT plate reader (BioTek, USA) at excitation and emission wavelengths of 360 and 460 nm, respectively.

### 4.7. DAPI/ PI Staining Assays

In this assay, PI (Sigma-Aldrich, Poole, UK) (1mg/mL) and DAPI (Sigma-Aldrich, UK) (1 mg/mL) were used to stain the cells of *S. aureus* (ATCC CRM 6538) and *E. coli* (ATCC CRM 8739). DAPI is a DNA binding dye staining all bacterial cells regardless of their viabilities, and PI is an ethidium derivative, which only can pass through damaged bacterial membranes and intercalates with nucleic acids, PI is one kind of vital stains to identify the ‘dead’ cells due to its characteristics mentioned above [62]. Bacteria were inoculated into TSB medium (Gram-positive bacteria) or LB medium (Gram-negative bacteria) at 37 °C overnight, and then sub-cultured at 37 °C for about 2.5 h. Subsequently, the cell cultures were centrifuged at 1000× *g* at 4 °C for 10 min to collect bacteria cells at the bottom. After the cells were washed twice with 5% TSB or 5% LB in 0.85% NaCl solution, they were resuspended with 5% TSB or 5% LB in 0.85% NaCl solution to obtain a cell concentration of 1 × 10^8^ CFU /mL by measuring the optical density (OD) value at 590 nm (Gram-positive bacteria: 0.68–0.72, Gram-negative bacteria: 0.65–0.7). Then, bacteria in logarithmic phase were incubated with peptide for 2 h, and then were centrifuged to collect bacterial cell pellets, which were then stained with PI for 15 min. After unbound PI was washed away by PBS, the cells were stained with DAPI for 15 min. Bacterial cultures without peptides, were considered as negative controls. The stained bacterial cells were examined using a Leica DMi8 fluorescence microscope (Leica, Wetzlar, Germany).

### 4.8. Antiproliferative Assays

The proliferative inhibition effect of GL-29 and its analogues were assessed by MTT assay as described in a previous study [63]. HaCaT cell line (human keratinocytes) and four cancer cell lines, U251MG (human glioblastoma astrocytoma cell line), HCT116 (human colon cancer cell line), H157 (human non-small lung cancer cell line) and H838 (human lung carcinoma cancer cell line), were used in this assay. A total of 8000 cells/per well were seeded in the 96-well plate for 24 h and then treated with peptides at the concentration from 100 μM to 100 nM for 24 h. In this assay, 1% Triton X-100 and PBS were set as a positive and negative control, respectively. After which, the formazan was dissolved in DMSO and the OD value was detected by use of a Synergy HT plate reader (BioTek, Minneapolis, MN, USA) at λ = 570 nm.

### 4.9. Haemolysis Assays

The haemolysis assays were performed by incubating prewashed erythrocyte suspension obtained from fresh defibrinated horse blood (TCS Biosciences Ltd., Buckingham, UK) with a range of peptides at final concentrations from 512 to 1 μM, for 2 h at 37 °C. The red cell suspensions were washed with phosphate-buffered saline (PBS) until the supernatant was clear and then diluted with PBS to form a 2% cell suspension. Peptides were 2-fold diluted by the 2% cell suspension to reach the final concentration range from 512 to 1 μM. The haemolysis of the cell suspensions with either 1% (*v/v*) Triton X-100 or PBS, were considered as positive controls and negative controls, respectively. After incubation, the extent of haemolysis was determined by measuring the absorbance of the supernatant from each sample at 570 nm with a Synergy HT plate reader (BioTek, USA).

### 4.10. Assessing the Efficacy of Peptides against S. aureus In Vivo

In this assay, *Galleria mellonella* larvae (waxworms) (Livefood UK Ltd., Rooks Bridge, UK) were used as an infection model to investigate the antimicrobial potency of peptides against *S. aureus*, which was described in a previous study in detail (Yuan et al., 2019). The larvae, weights between 225 mg and 275 mg, were selected, and nine larvae were stored in a separate Petri dish as a group and they were divided into several groups. Then, 10 µL of the peptide solution at final concentrations of 8 µM and 16 µM, respectively, and 10 µL of PBS were injected into larvae to test the toxicity of peptides and PBS solution. Afterwards, 10 μL of the bacterial suspension in PBS (5 × 10^7^ CFU/mL) were injected into larvae. All the infected larvae were ensured to be alive after 2 h of the injection, followed by the treatment with 10 μL of the peptide solution to reach final concentrations of 2, 4 and 8 μM, respectively. The negative control was treated with the same volume of PBS, and the positive control was dosed with the same volume of 20 mg/kg vancomycin (Sigma-Aldrich, UK). The number of living larvae was counted every 24 h for 5 days, except the first day of 12 h.

### 4.11. Statistical Analysis

Data were analysed using Prism (Version 8.2.1; GraphPad Software, Inc, San Diego, CA, USA). Error bars in the graphs represented standard errors of means (SEM) of nine repeats from three independent tests. The statistical significance was calculated using two-way ANOVA and is indicated as ns (non-significant difference), * (*p* < 0.05), ** (*p* < 0.01), *** (*p* < 0.001), ****(*p* < 0.0001). The HC_10,_ HC_50_ and IC_50_ values were calculated using the online tool AAT Bioquest (https://www.aatbio.com/index.html, accessed on 6 May 2022).

## 5. Conclusions

In conclusion, palustrin-2 peptides were identified as antimicrobial peptides with broad-spectrum antimicrobial activity and low haemolysis, and palustrin-2ISb was a typical representative according to previous studies. In this study, both the structures of disulphide bridge and the ‘Rana box’ itself, were discovered to have a negligible effect on the antimicrobial activity and the removal of this motif reduced haemolysis and cytotoxicity against HaCaT. In light of the further studies on GL-22, the N-terminal region before glycine and the C-terminal region after glycine, exhibited very weak, or no, antimicrobial activity, respectively. Since the N-terminal segment of some other cationic AMPs was reported to exhibit similar bioactivity as the intact AMPs [25], the reasons why the N-terminal section of GL-22 was inactive needs to be further studied.

## Figures and Tables

**Table 1:** Physicochemical parameters of GL-29 and its truncated analogues.

Peptide	Sequence	Net Charge(z)	Hydrophobic Moment (μH)	Hydrophobicity (H)
GL-29	GLWNSIKIAGKKLFVNVLDKIRCKVAGGC	+5	0.244	0.480
GL-22	GLWNSIKIAGKKLFVNVLDKIR-NH_2_	+5	0.402	0.469
GL-9	GLWNSIKIA-NH_2_	+2	0.403	0.692
LF-10	LFVNVLDKIR-NH_2_	+2	0.653	0.606
FV-9	FVNVLDKIR-NH_2_	+2	0.739	0.484
VN-8	VNVLDKIR-NH_2_	+2	-	-
FV-8	FVNVLDKI-NH_2_	+2	-	-

The predicted net charge, hydrophobic moment and hydrophobicity were shown in the table according to different sequences. The physical parameter of VN-8 and FV-8 cannot be analysed by Heliquest due to the short length of the sequence.

**Table 2:** The MICs and MBCs of GL-29 and its short analogues.

Microorganisms	MIC/MBC (μM)						
	GL-29	GL-22	GL-9	LF-10	FV-9	FV-8	VN-8
*S. aureus* (ATCC CRM 6538)	4/4	4/4	512/512	>512	>512	>512	>512
*Enterococcus faecalis* (*E. faecalis*) (NCTC 12697)	16/32	32/32	>512	>512	>512	>512	>512
MRSA (ATCC CRM 12493)	8/8	8/8	>512	>512	>512	>512	>512
*E. coli* (ATCC CRM 8739)	2/4	4/4	512/>512	>512	>512	>512	>512
*K. pneumoniae* (ATCC CRM 43816)	8/8	4/4	>512	>512	>512	>512	>512
*Pseudomonas aeruginosa* (*P. aeruginosa*) (ATCC CRM 9027)	16/16	32/32	>512	>512	>512	>512	>512
*C. albicans* (ATCC CRM 10231)	128/256	128/256	>512	>512	>512	>512	>512
Clinical isolated strains							
*S. aureus* (B042 V2E1 A)	4/4	32/64	>512	>512	>512	>512	>512
MRSA (B038 V1S1 A)	2/2	4/4	>512	>512	>512	>512	>512
KPC-producing resistant strains							
*E. coli* (ATCC BAA-2340)	2/4	2/4	>512	>512	>512	>512	>512
*K. pneumoniae* (ATCC BAA-1705)	4/4	4/4	>512	>512	>512	>512	>512
HC_50_ (μM)	33.03	254.11	-	-	-	-	-
HC_10_ (μM)	5.86	87.304	-	-	504.21	504.21	-
IC_50_ (μM) (HaCaT)	5.05	38.90	-	-	-	-	-

The table represents the antimicrobial results of 15 replicates from three independent assays. HC_50_ and HC_10_ values are the peptide concentrations that produces 50% and 10% haemolysis of horse erythrocytes. “-” means the HC_50_, HC_10_ or IC_50_ were not detected in this study. KPC represents *Klebsiella pneumoniae* carbapenemase.

**Table 3:** The MBICs and MBECs of GL-29 and its analogues against reference bacteria.

Peptides	MBIC/MBEC (μM)					
	*S. aureus* (ATCC CRM 6538)	*E. faecalis* (NCTC 12697)	MRSA (ATCC CRM 12493)	*E. coli* (ATCC CRM 8739)	*K. pneumoniae* (ATCC CRM 43816)	*P. aeruginosa* (ATCC CRM 9027)
GL-29	4/128	16/>512	8/256	4/128	16/>512	64/>512
GL-22	8/>512	32/>512	8/>512	16/>512	16/>512	128/>512
GL-9	>512	>512	>512	512/>512	>512	>512
LF-10	>512	>512	>512	>512	>512	>512
FV-9	>512	>512	>512	>512	>512	>512
FV-8	>512	>512	>512	>512	>512	>512
VN-8	>512	>512	>512	>512	>512	>512

The table represents the antibiofilm results of 15 replicates from three independent assays.

**Table 4:** The IC_50_ values of GL-29 and GL-22 against different cell lines.

Human Cell Lines	IC_50_ (μM)			
	U251MG	HCT116	H157	H838
GL-29	6.60	17.86	1.74	5.38
GL-22	40.58	128.89	9.36	25.25

## Data Availability

Not applicable.

## Supplementary Materials

The following supporting information can be downloaded at: https://www.mdpi.com/article/10.3390/antibiotics11081048/s1, Figure S1: Secondary structure prediction of (a) GL-29, (b) GL-22, (c) GL-9, (d) LF-10 and (e) FV-9; Figure S2: Helical wheel projections of (a) GL-29, (b) GL-22, (c) GL-9, (d) LF-10 and (e) FV-9 with the arrow indicating the direction of the hydrophobic moments; Figure S3: RP–HPLC chromatograph of GL-29 and its analogues at a wavelength of 214nm and the arrow indicates the retention times/elution position of the peptide; Figure S4: MALDI-TOF mass spectrum of GL-29 and its analogues; Table S1: Secondary structure analysis of GL-22 truncated analogues in different solutions.
