# Supplementary material for: Targeted Modification and Structure-Activity Study of GL-29, an Analogue of the Antimicrobial Peptide Palustrin-2ISb
Source: Antibiotics (Basel). 2022 Aug 3;11(8):1048. doi: 10.3390/antibiotics11081048 (PMC9405102; doi:10.3390/antibiotics11081048)
Supplement: Supplementary file 1 [file antibiotics-11-01048-s001.zip › antibiotics-1824329-supplementary.pdf]

Article

Targeted modification and structure-activity study of GL-29, an analogue of the antimicrobial peptide palustrin-2ISb

Siyan Liu, Yaxian Lin, Jiachen Liu, Xiaoling Chen, Chengbang Ma, Xinping Xi, Mei Zhou, Tianbao Chen[1], James F Burrows and Lei Wang

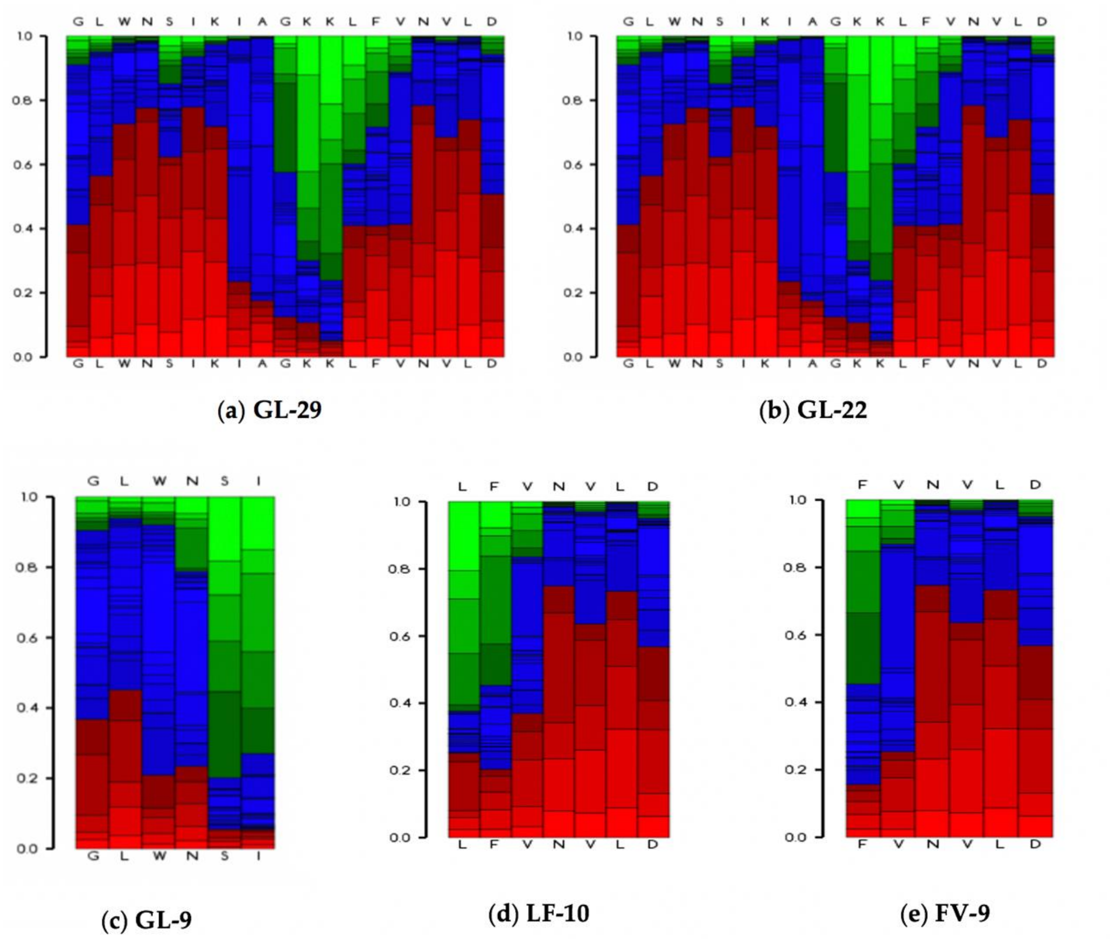

Figure S1: Secondary structure prediction of (a) GL-29, (b) GL-22, (c) GL-9, (d) LF-10 and (e) FV-9. The red, blue and green blocks in the figure represent the helical, coil and extended conformations. The secondary structure of VN-8 and FV-8 cannot be predicted due to the short length of the sequence.

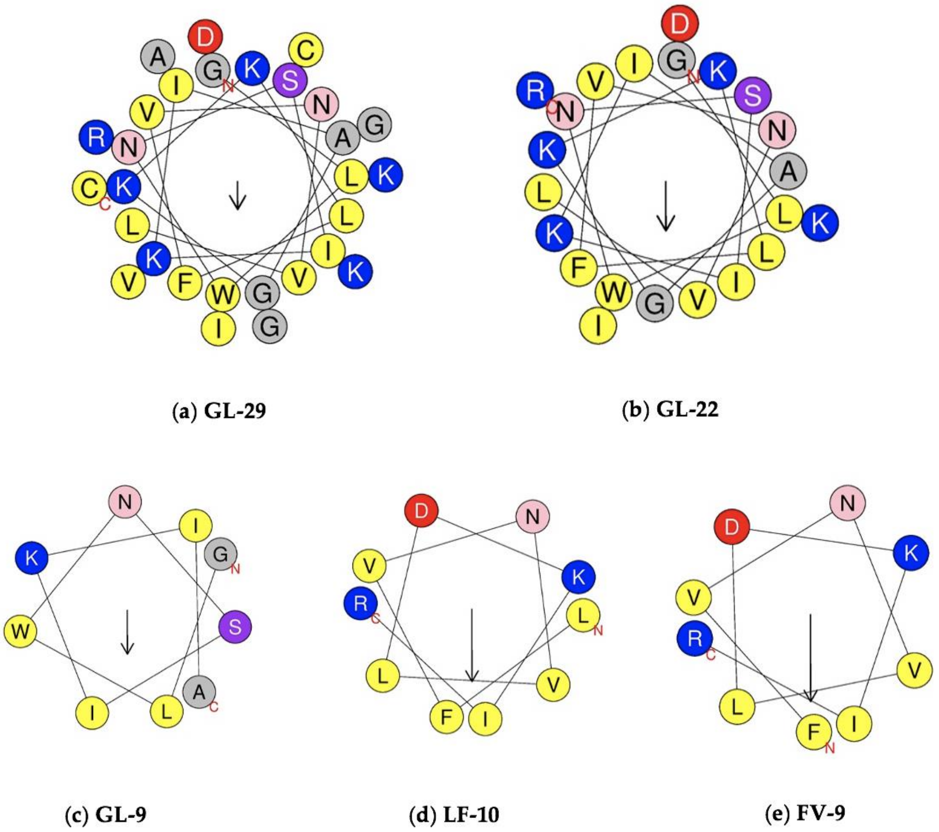

Figure S2: Helical wheel projections of (a) GL-29, (b) GL-22, (c) GL-9, (d) LF-10 and (e) FV-9 with the arrow indicating the direction of the hydrophobic moments. The physical parameter of VN-8 and FV-8 cannot be analysed by Heliquest due to the short length of the sequence.

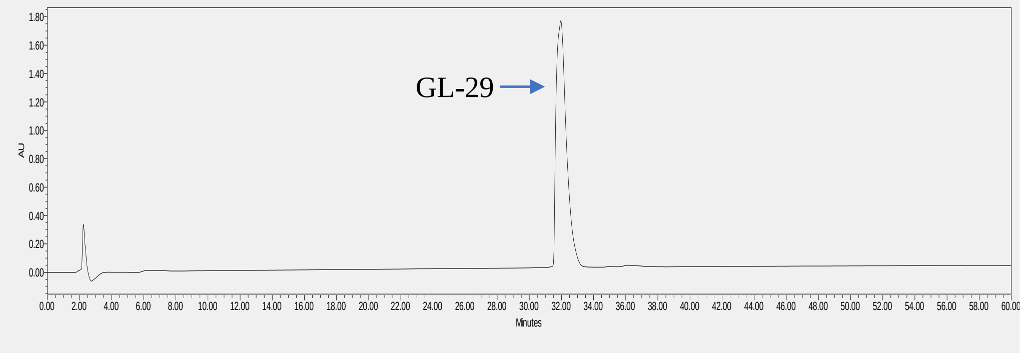

(a)

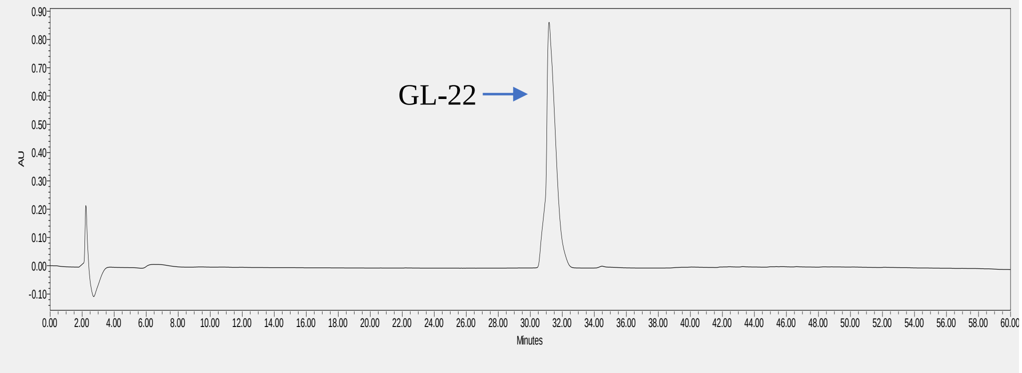

(b)

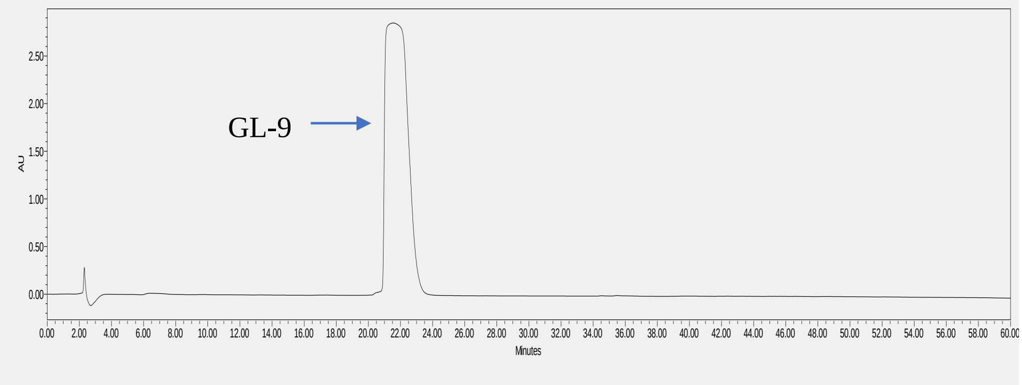

(c)

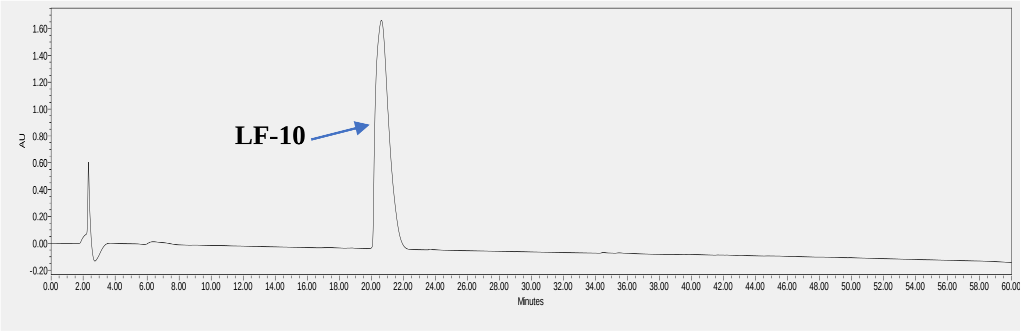

(d)

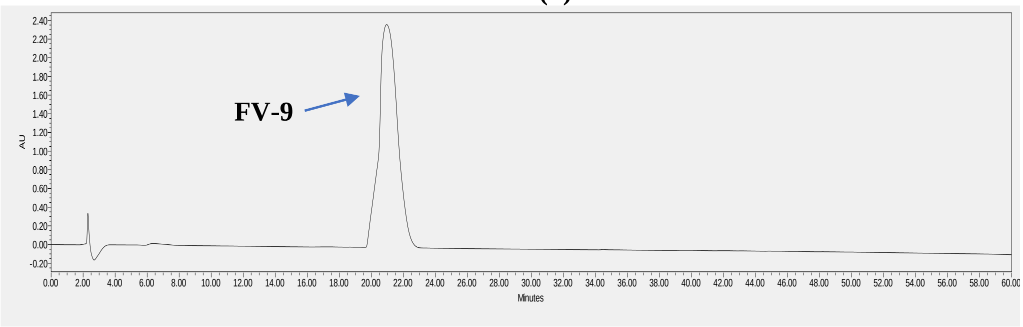

(e)

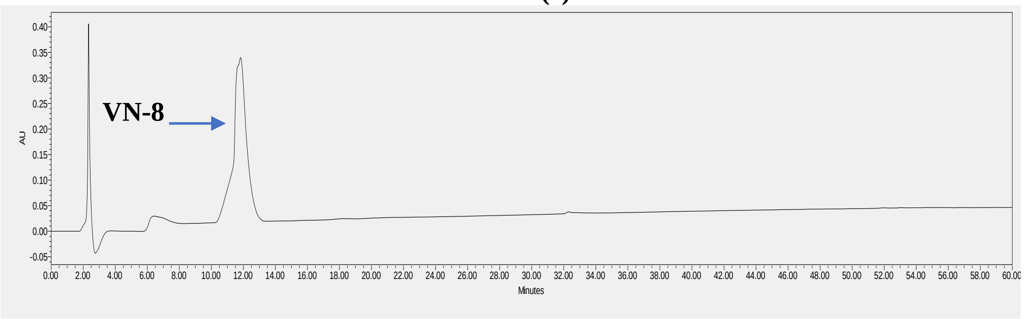

(f)

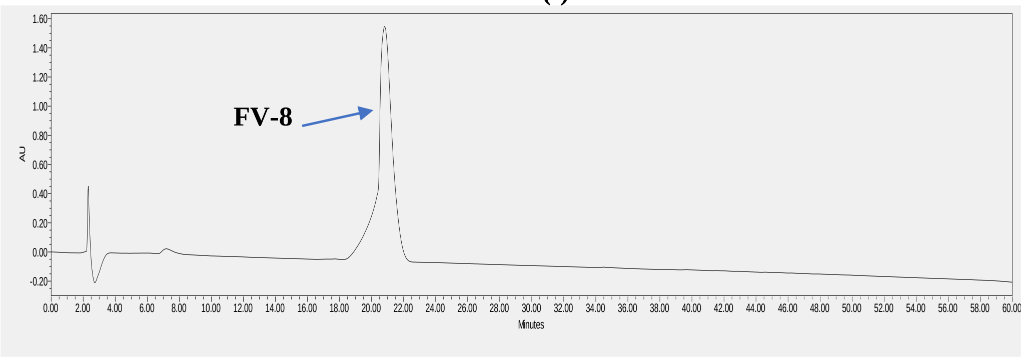

(g)

Figure S3: RP-HPLC chromatograph of GL-29 (**a**) and its analogues (**b-g**) at wavelength of 214nm and the arrow indicates the retention times/elution position of the peptide.

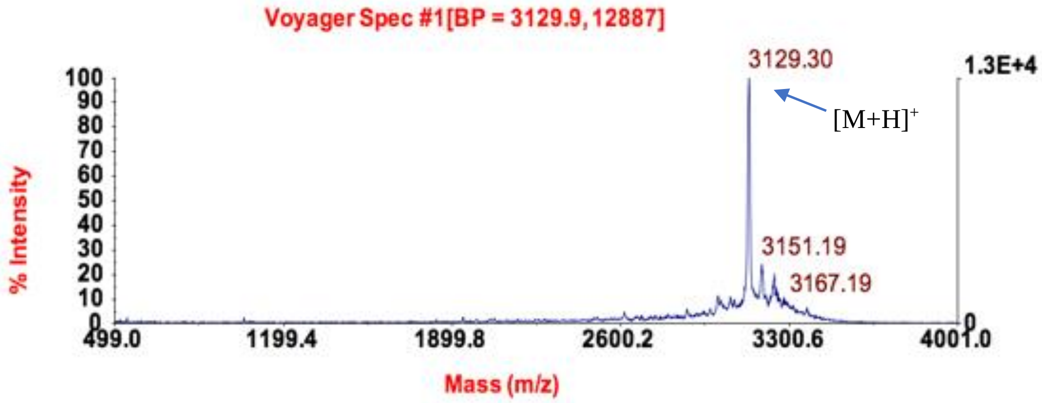
Voyager Spec #1[BP = 3129.9, 12887]
3129.30
1.3E+4
[M+H]+
3151.19
3167.19
% Intensity
100
90
80
70
60
50
40
30
20
10
0
499.0
1199.4
1899.8
2600.2
3300.6
4001.0
0
Mass (m/z)

(a)

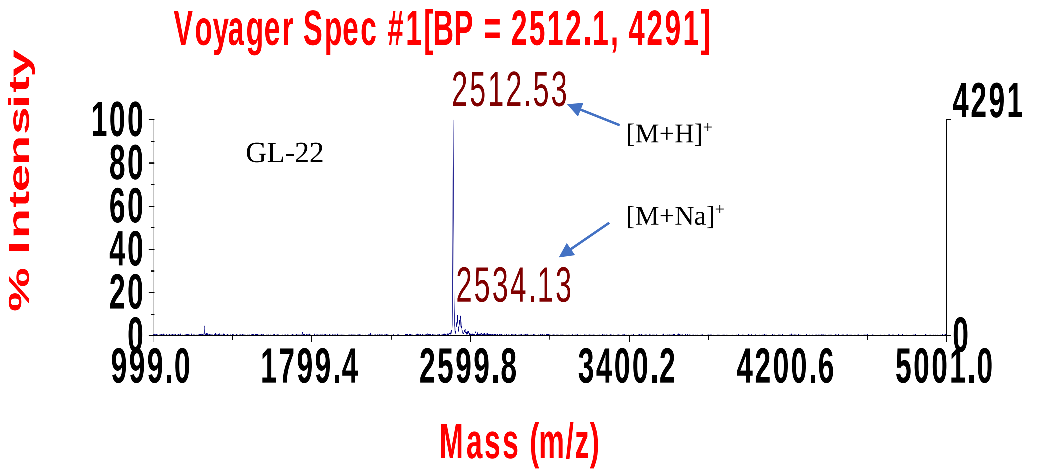
Voyager Spec #1[BP = 2512.1, 4291]
2512.53
4291
[M+H]+
GL-22
[M+Na]+
2534.13
% Intensity
100
80
60
40
20
0
999.0
1799.4
2599.8
3400.2
4200.6
5001.0
0
Mass (m/z)

(b)

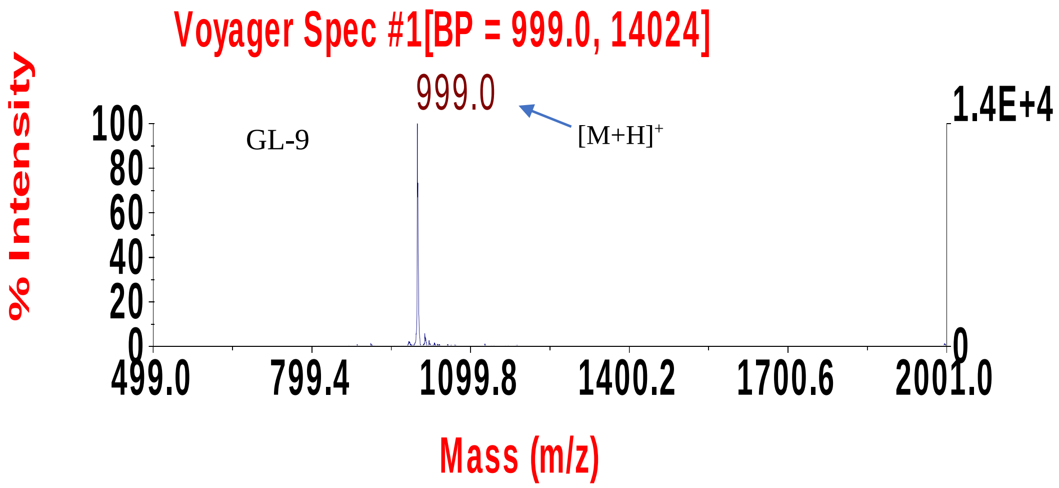
Voyager Spec #1[BP = 999.0, 14024]
999.0
1.4E+4
[M+H]+
GL-9
% Intensity
100
80
60
40
20
0
499.0
799.4
1099.8
1400.2
1700.6
2001.0
0
Mass (m/z)

(c)

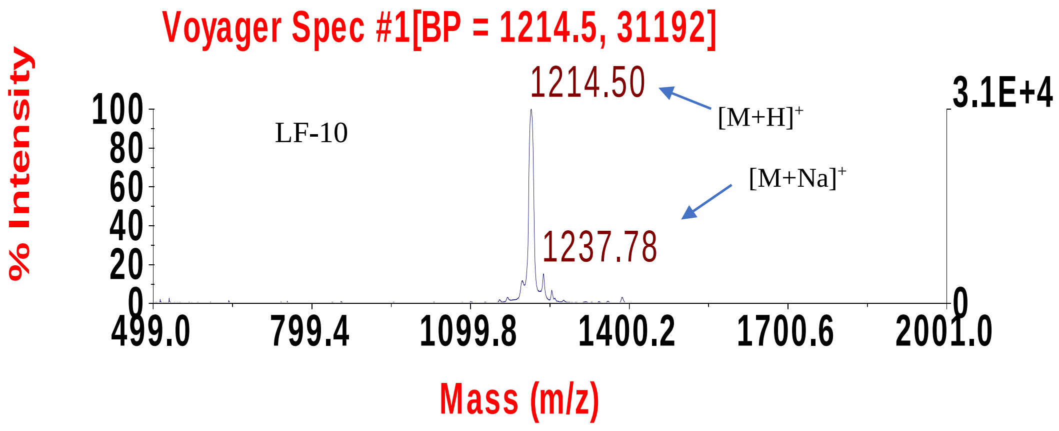
Voyager Spec #1[BP = 1214.5, 31192]
1214.50
[M+H]+
[M+Na]+
1237.78
LF-10
% Intensity
100
80
60
40
20
0
3.1E+4
0
499.0
799.4
1099.8
1400.2
1700.6
2001.0
Mass (m/z)

(d)

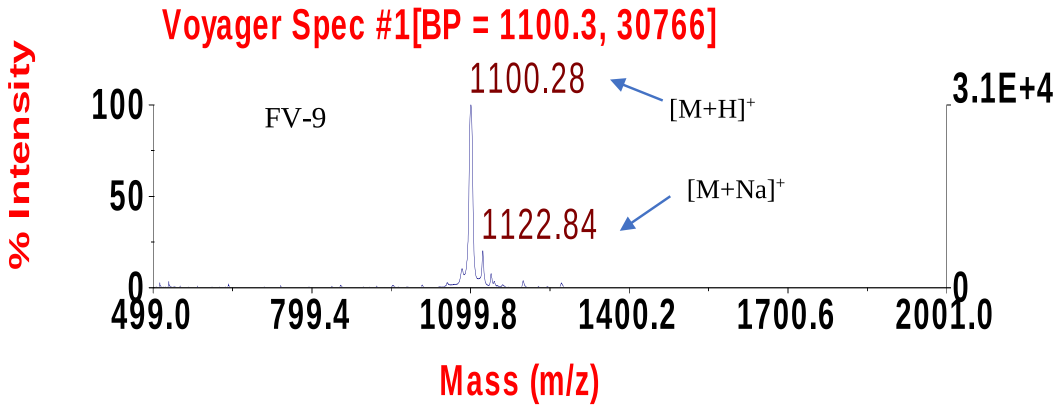
Voyager Spec #1[BP = 1100.3, 30766]
1100.28
[M+H]+
[M+Na]+
1122.84
FV-9
% Intensity
100
50
0
3.1E+4
0
499.0
799.4
1099.8
1400.2
1700.6
2001.0
Mass (m/z)

(e)

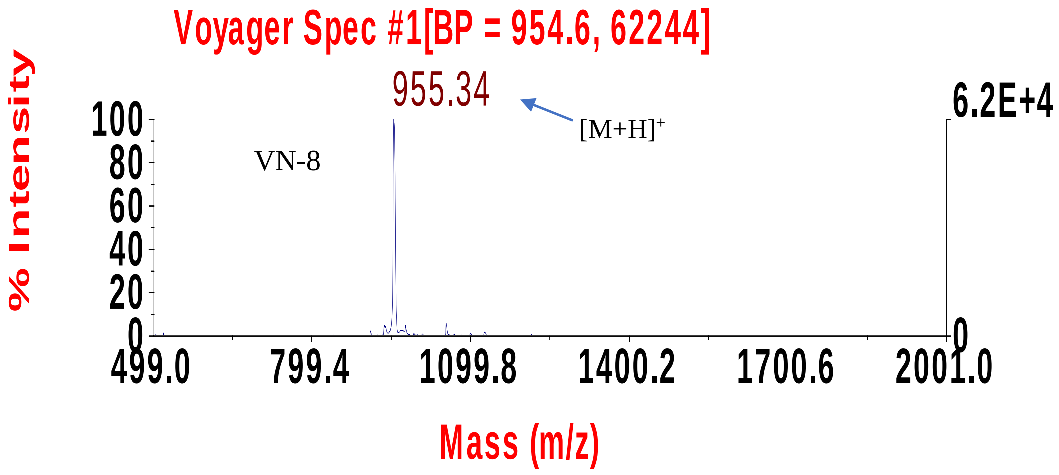
Voyager Spec #1[BP = 954.6, 62244]
955.34
[M+H]+
VN-8
% Intensity
100
80
60
40
20
0
6.2E+4
0
499.0
799.4
1099.8
1400.2
1700.6
2001.0
Mass (m/z)

(f)

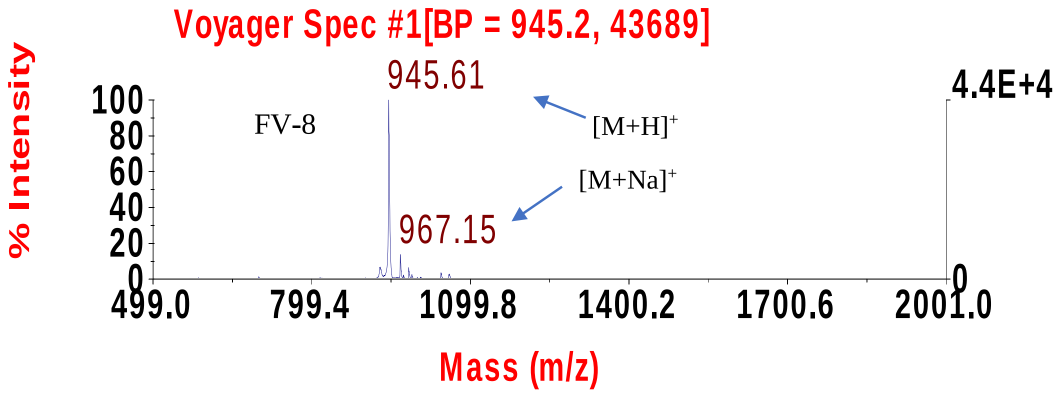

(g)

Figure S4: MALDI-TOF mass spectra of GL-29 and its analogues. MALDI-TOF mass spectrum of GL-29, the $[M+H]^+$ ion was observed as 3129.30 Da and the peak with a mass of 3151.19 Da and 3167.19 Da represent the sodium adductive ion $[M+Na]^+$ and the potassium adductive ion $[M+K]^+$, respectively (**a**). MALDI-TOF mass spectrum of GL-22, the $[M+H]^+$ ion was observed as 2512.54 Da and the peak with a mass of 2534.56 Da represents the sodium adductive ion $[M+Na]^+$ (**b**). MALDI-TOF mass spectrum of GL-9, the $[M+H]^+$ ion was observed as 999.0 Da (**c**). MALDI-TOF mass spectrum of LF-10, the $[M+H]^+$ ion was observed as 1214.50 Da and the peak with a mass of 1237.78 Da represents the sodium adductive ion $[M+Na]^+$(**d**). MALDI-TOF mass spectrum of FV-9, the $[M+H]^+$ ion was observed as 1100.28 Da and the peak with a mass of 1122.84 Da represents the sodium adductive ion $[M+Na]^+$ (**e**). MALDI-TOF mass spectrum of VN-8, the $[M+H]^+$ ion was observed as 955.34 Da (**f**). MALDI-TOF mass spectrum of FV-8, the $[M+H]^+$ ion was observed as 945.61 Da and the peak with a mass of 967.15 Da represents the sodium adductive ion $[M+Na]^+$ (**g**).

Table S1: Secondary structure analysis of GL-22 truncated analogues in different solutions. These data represent the percentage (%) of helix, antiparallel and turn contents of different peptides. These were analysed by BeStSel (http://bestsel.elte.hu/index.php).

Peptide	H_2O			50%TFE		
	Helix	Antiparallel	Turn	Helix	Antiparallel	Turn
GL-29	6.0	28.1	15.7	49.4	0.4	12.7
GL-22	3.6	27.5	16.3	50	4.8	11.7
GL-9	8.4	28.6	13.9	23.0	23.1	14.6
LF-10	11.6	20.3	17.1	41.6	9.3	10.6
FV-9	7.7	43.6	7.7	45.2	4.7	0.0
FV-8	10.8	17	18.4	18.5	32.8	14.3
VN-8	3.4	34.2	14.3	24.0	28.9	12.3
